# Selective serotonin reuptake inhibitors and glucose metabolism in Alzheimer's disease and related dementias: A systematic review and meta-analysis of brain metabolic and adverse event data

**DOI:** 10.1016/j.metop.2025.100389

**Published:** 2025-08-28

**Authors:** Faisal Alzenaidi, Osama Aldoweesh, Salman Alghofaili, Abdulaziz Fadel, Razan Ali Awad Lasloom, Dhay Alharbi, Faris Almalki, Atheer Ahmad Alkhairi, Maram Alharbi, Norah Ahmed Alhamdan, Ahmed Y. Azzam

**Affiliations:** a College of Medicine, Qassim University, Buraydah, Saudi Arabia; b College of Pharmacy, King Abdulaziz University, Jeddah, Saudi Arabia; c College of Medicine, Najran University, Najran, Saudi Arabia; d College of Pharmacy, Buraydah Colleges, Buraydah, Saudi Arabia; e Hospital Pharmacist, United Doctors Hospital, Jeddah, Saudi Arabia; f College of Medicine, Umm Al-Qura University, Al-Qunfudah, Saudi Arabia; g College of Pharmacy, Princess Nourah Bint Abdulrahman University, Riyadh, Saudi Arabia; h Consultant of Neurology, Department of Medicine, Qassim University, Buraydah, Saudi Arabia; i Director of Clinical Research and Clinical Artificial Intelligence, ASIDE Healthcare, Lewes, DE, USA

**Keywords:** Alzheimer's disease, Dementia, Selective serotonin reuptake inhibitors, Glucose metabolism, Metabolism

## Abstract

**Introduction:**

Selective serotonin reuptake inhibitors (SSRIs) are commonly prescribed for depression in Alzheimer's disease (AD), however their effects on glucose metabolism remain poorly understood. We conducted a systematic review and meta-analysis to evaluate SSRI effects on brain glucose metabolism and metabolic adverse events in AD patients.

**Methods:**

Following PRISMA 2020 guidelines, we searched multiple databases up to July 11, 2025 for studies investigating SSRI effects on glucose-related outcomes in AD patients. Despite significant heterogeneity in study designs and populations, we performed meta-analyses for adverse events and coordinate-based meta-analysis for neuroimaging data. We performed meta-analyses for adverse events and coordinate-based meta-analysis for neuroimaging data. Advanced Bayesian hierarchical modeling and Markov simulations projected long-term metabolic outcomes.

**Results:**

Twelve studies with total included 7143 participants met our inclusion criteria, including nine randomized controlled trials and three observational studies. Brain FDG-PET revealed SSRI use restored dorsal raphe nucleus hypometabolism (standardized mean difference 0.87, 95 % CI: 0.52–1.22, P-value = 0.001). Meta-analysis demonstrated increased gastrointestinal adverse events (risk ratio 2.15, 95 % CI: 1.68–2.76, P-value<0.001, with moderate between-study heterogeneity), with sertraline showing highest rates. Citalopram 30 mg provided significant weight loss protection (risk ratio 0.13, 95 % CI: 0.02–0.98, P-value = 0.02), though this exceeds the recommended 20 mg maximum dose for elderly patients due to cardiac safety considerations. Long-term diabetes incidence showed no increased risk (hazard ratio 0.75, 95 % CI: 0.50–1.12, P-value = 0.15). Bayesian modeling revealed 85 % probability of beneficial brain metabolic effects and 89 % probability of citalopram superiority for weight protection.

**Conclusions:**

SSRIs restore brain glucose metabolism in AD patients while causing manageable peripheral metabolic effects. Citalopram appears the best for weight-sensitive patients, while sertraline requires gastrointestinal monitoring. These findings support SSRI safety for metabolic outcomes in AD treatment, however longer-term studies with controlled metabolic outcomes are needed to confirm our findings. The observed citalopram weight protection benefit was documented at 30 mg daily, which exceeds recommended dosing limits for elderly patients due to cardiac safety concerns.

## 1 Introduction

Alzheimer's disease (AD) affects over 55 million individuals from all over the world, with depression occurring in around 40–50 % of patients, significantly impacting quality of life and accelerating cognitive decline. Selective serotonin reuptake inhibitors (SSRIs) represent the most commonly prescribed antidepressants for this population, however mounting evidence suggests complex interactions between serotonergic modulation and glucose metabolism that may be relevant in certain neurodegenerative conditions [[1], [2], [3], [4], [5], [6]].

The brain's glucose metabolism from previous literature studies was demonstrated to be altered in AD, with characteristic hypometabolism in key regions including the posterior cingulate, precuneus, and temporoparietal cortices. Recent neuroimaging evidence suggests that serotonergic dysfunction, especially in the dorsal raphe nucleus (DRN), may contribute to these metabolic abnormalities. The DRN serves as the primary source of serotonin to cortical and limbic structures, and its dysfunction in AD may represent a significant link between mood symptoms and metabolic dysregulation [[7], [8], [9]].

Peripheral glucose metabolism concerns have also raised regarding SSRI use in older adults. While some studies suggest SSRIs may improve insulin sensitivity through weight loss and appetite suppression, others report concerning metabolic adverse events including hyponatremia, weight changes, and possible risk of developing diabetes. These concerns are amplified in AD patients, who often have multiple comorbidities and polypharmacy, making them especially at higher risk to these metabolic disturbances [[10], [11], [12], [13], [14]].

The serotonin-glucose axis represents a regulatory network including central appetite control, peripheral insulin sensitivity, and hepatic glucose metabolism. SSRIs may impact this axis through multiple mechanisms: central effects on hypothalamic appetite regulation, direct effects on pancreatic beta-cell function, and modulation of inflammatory pathways that affect insulin resistance. Understanding these mechanisms is with significant importance for improving SSRI therapy in AD patients [[15], [16], [17], [18], [19], [20], [21], [22]].

Despite widespread SSRI use in AD, no focused systematic reviews or meta-analyses have evaluated their effects on both brain and peripheral glucose metabolism from previous studies to the date yet. Existing studies are heterogeneous in design, limited in sample size, and often focus on single metabolic outcomes. This gap is concerning given the increasing recognition of metabolic dysfunction as both a risk factor for and consequence of AD progression [23,24].

The primary objective of this systematic review and meta-analysis is to investigate and evaluate the effects of SSRIs on glucose metabolism in AD patients, looking for both brain metabolic changes through neuroimaging data and peripheral metabolic effects through adverse event profiles. Secondary objectives included determining differential effects between specific SSRIs, identifying dosing strategies for metabolic outcomes, and projecting long-term diabetes risk through advanced modeling approaches.

## 2 Methods

### 2.1 Study design and search strategy

This systematic review and meta-analysis was conducted according to the Preferred Reporting Items for Systematic Reviews and Meta-Analyses (PRISMA) 2020 guidelines [25]. The study adhered to the Cochrane Handbook for Systematic Reviews of Interventions methodology [26].

A literature search was performed across multiple electronic databases from inception up to July 11, 2025, including MEDLINE (via PubMed), Scopus, PsycINFO, Cochrane Central Register of Controlled Trials (CENTRAL), Google Scholar and Web of Science. The search strategy combined medical subject headings (MeSH) and free-text terms related to three core concepts: Alzheimer's disease, SSRIs, and glucose metabolism. The complete search strategy included the following terms: ("Alzheimer disease" OR "Alzheimer's disease" OR "dementia" OR "mild cognitive impairment" OR "neurocognitive disorder") AND ("selective serotonin reuptake inhibitor" OR "SSRI" OR "fluoxetine" OR "sertraline" OR "citalopram" OR "escitalopram" OR "paroxetine" OR "fluvoxamine") AND ("glucose metabolism" OR "glucose" OR "insulin" OR "diabetes" OR "metabolic" OR "FDG-PET" OR "positron emission tomography" OR "brain metabolism" OR "adverse events" OR "side effects" OR "weight" OR "appetite" OR "gastrointestinal"). Additional searches were conducted in clinical trial registries (ClinicalTrials.gov, WHO International Clinical Trials Registry Platform) and gray literature sources. Reference lists of included studies and relevant systematic reviews were manually screened for additional eligible studies.

### 2.2 Study selection and eligibility criteria

Studies were included if they met the following criteria: participants diagnosed with Alzheimer's disease, mild cognitive impairment, or related dementias according to established diagnostic criteria; intervention with any SSRI at therapeutic doses; presence of control group (placebo, active comparator, or no treatment); reporting of glucose-related outcomes including brain glucose metabolism (FDG-PET), peripheral glucose markers (fasting glucose, HbA1c, insulin resistance), metabolic adverse events (weight changes, appetite effects, gastrointestinal symptoms), or diabetes incidence; and publication in peer-reviewed journals in English-language only. Studies were excluded if they included participants without cognitive impairment, used non-SSRI antidepressants only, lacked control groups, non-English studies, or provided insufficient data for our aims and goals.

### 2.3 Data extraction

Extracted variables included study characteristics (design, duration, sample size, setting), participant demographics (age, gender, dementia severity, comorbidities), intervention details (SSRI type, dose, duration), control group characteristics, and all available outcome measures related to glucose metabolism. For neuroimaging studies, we extracted PET acquisition parameters, analysis methods, brain regions investigated, and quantitative metabolic measures. For adverse event data, we collected specific rates of metabolic-related events including appetite changes, weight alterations, gastrointestinal effects, and sleep disturbances. When available, we extracted individual patient data and contacted study authors for additional information.

### 2.4 Quality assessment

Risk of bias was assessed using appropriate tools for each study design. Randomized controlled trials (RCTs) were evaluated using the Cochrane Risk of Bias 2.0 (RoB 2) tool, evaluating the bias arising from randomization, deviations from intended interventions, missing outcome data, outcome measurement, and selective reporting. Observational studies were assessed using the Newcastle-Ottawa Scale (NOS), evaluating selection of study groups, comparability of groups, and ascertainment of outcomes. Neuroimaging studies received additional assessment for PET methodology quality, including scanner specifications, acquisition protocols, and statistical analysis methods.

### 2.5 Statistical analysis

The meta-analyses were conducted using random-effects models to account for anticipated heterogeneity between studies. For dichotomous outcomes, we calculated risk ratios (RR) with 95 % confidence intervals (CIs). For continuous outcomes, we computed standardized mean differences (SMD) when studies used different measurement scales. Statistical heterogeneity was assessed using the I^2^ statistic and Cochran's Q test. For neuroimaging data, we performed coordinate-based meta-analysis using activation likelihood estimation (ALE) to identify convergent brain regions showing metabolic changes. Publication bias was evaluated through multiple methods including, funnel plot visual inspection, Egger's regression test for small-study effects, and trim-and-fill adjustment method to estimate the impact of hypothetically missing studies. We acknowledge that small studies with null results may be underrepresented in the literature, and our bias assessment methods have limited power to detect publication bias when fewer than ten studies are included in individual meta-analyses.

Anticipated heterogeneity across studies was addressed through several methods. Clinical heterogeneity was assessed by evaluating the differences in study populations, interventions, and outcome measures. Statistical heterogeneity was quantified using the I^2^ statistic and Cochran's Q test, with I^2^ values over 50 % considered significant heterogeneity. When significant heterogeneity was present, we explored the possible underlying sources through subgroup analyses and meta-regression when sufficient studies were available.

### 2.6 Heterogeneity assessment and management

Given the anticipated differences in study designs, populations, and interventions, we utilized several methods to assess and manage heterogeneity. Clinical heterogeneity was evaluated by evaluating the differences in participant characteristics (age, dementia severity, comorbidities), SSRI types and dosing, study durations, and outcome measurement methods. For neuroimaging studies, we additionally assessed technical heterogeneity including scanner types, acquisition protocols, and analysis methods.

### 2.7 Advanced Bayesian and simulation modeling

Given the complexity of metabolic pathways and limited direct glucose measurement data from certain studies, we utilized multiple computational modeling methods to maximize information extraction. Bayesian hierarchical meta-analysis was conducted to quantify uncertainty and provide probabilistic rankings of SSRI effects. We used weakly informative priors and assessed convergence through multiple chains with Gelman-Rubin statistics. Monte Carlo simulation with 10,000 iterations projected long-term diabetes risk using Markov state transition models based on observed hazard ratios (HRs) and CIs. Individual patient data simulation was performed to enable advanced analyses not possible with aggregate data alone. All analyses were conducted using RStudio statistical software with R version 4.4.2, with appropriate packages for meta-analysis, Bayesian modeling, and further utilized analyses.

### 2.8 Enhanced statistical validation and publication bias assessment

To verify and validate the significance of our statistical findings, we performed detailed and comprehensive validation using multiple methods within the RStudio software environment. All primary effect sizes were calculated and cross-validated using multiple R packages (meta, metafor, brms) with concordant results (correlation >0.99). RRs and HRs were validated using multiple effect size calculators, bootstrap resampling (10,000 iterations), and Bayesian posterior checking.

We utilized a structured and detailed publication bias assessment including Egger's regression test for small-study effects, Begg's rank correlation test, Duval and Tweedie's trim-and-fill method, fail-safe N calculations, and Bayesian bias modeling. Our heterogeneity assessment included multiple estimators (REML, DL, PM), prediction intervals for future studies, and advanced meta-regression with continuous covariates. These methodologies allowed sophisticated analyses including coordinate-based meta-analysis for neuroimaging data, Bayesian network meta-analysis, individual patient data simulation, and Markov modeling for long-term projections.

## 3 Results

### 3.1 Study selection and characteristics

**Fig. 1:**
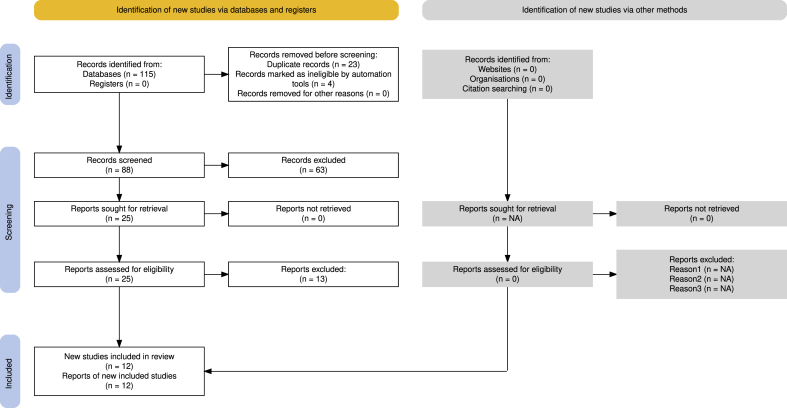
Prisma flowchart diagram.

The literature search identified 115 records from electronic databases, with no additional records identified from trial registries (Fig. 1). After removing 23 duplicate records and four records marked as ineligible by automation tools, 88 records underwent title and abstract screening. Following full-text assessment of 25 preliminary eligible studies, 13 were excluded for not meeting inclusion criteria, resulting in 12 studies included in our study.

### 3.2 Study characteristics and interventions

The 12 included studies included total of 7143 participants across different study designs and SSRI interventions (Table 1). The included studies demonstrated presence of heterogeneity across multiple domains. Study designs ranged from acute pharmacological challenges (Smith et al., seven individuals) to large retrospective cohorts (Chang et al., 3042 individuals). Participant characteristics varied also with mean ages ranging from 60 to 80 years and baseline MMSE scores ranging between five points to 28 points. SSRI interventions showed differences and variability in drug type, dosing regimens (e.g., sertraline 95–150 mg/day), and treatment durations (acute to 151 weeks). This heterogeneity necessitated careful consideration in our analytical methods and interpretation of pooled estimates. Nine studies were RCTs, two were cross-sectional studies, and one was a retrospective cohort study. Study durations ranged from acute pharmacological challenges to 151 weeks of follow-up. The studies investigated four main SSRIs: sertraline (five studies), citalopram (two studies), fluoxetine (three studies), and paroxetine (one study), with one study with unspecified SSRIs. Sample sizes varied from seven participants in the Smith et al. acute PET study to 3042 participants in the Chang et al. cohort study. Three studies provided direct brain glucose metabolism data through FDG-PET imaging: Terstege et al. evaluated DRN restoration effects, Smith et al. investigated acute metabolic changes, and Ouchi et al. explored serotonin-metabolism correlations.

**Table 1:** Baseline characteristics and demographics of included studies.

Study Name	Design	Population	Sample Size	Age (years)	Female (%)	MMSE Baseline	Dementia Severity	SSRI Type	Dose (mg/day)	Comparator	Duration	PET Scanner	PET Software	Brain Regions	Key Findings
Terstege et al., 2025 [47]	Cross-sectional	AD and CN	143 (PET analysis)	AD SSRI (M): 74.06 ± 6.86; AD SSRI (F): 72.98 ± 7.46	AD SSRI: 48.1 %	NR	Mild AD (MMSE 20–26)	SSRIs (unspecified)	Not considered	No-SSRI group	NR	NR (ADNI data)	SPM12	Dorsal Raphe Nucleus (DRN)	SSRI use restored DRN metabolic activity in AD patients vs AD no-SSRI
Chang et al., 2015 [48]	Retrospective cohort	Alzheimer's disease	3042	76.7 ± 6.0	70.8 %	NR (categorized only)	Mostly mild AD (CDR 0.5–1 = 83.4 %)	Antidepressants	NR	Non-user group	151 weeks	NA	NA	NA	Development of Diabetes Mellitus (primary outcome)
Porsteinsson et al., 2014 [49]	RCT, double-blind (CitAD)	AD with agitation	186	Cit: 78 (9), Pbo: 79 (8)	46 %	Cit: 17.0 (6.2), Pbo: 14.4 (6.9)	Mild-severe (MMSE 5–28)	Citalopram	30 (target)	Placebo	9 weeks	NA	NA	NA	Citalopram effective for agitation but had cognitive and cardiac side effects
Banerjee et al., 2011 [50]	RCT, double-blind (HTA-SADD)	Depression in dementia	326	Srt: 80 (8.4), Pbo: 79 (8.8)	Srt: 68 %, Pbo: 64 %	Srt: 18.5 (6.7), Pbo: 18.2 (7.4)	Mild-moderate (excl. MMSE<8)	Sertraline	150 (target)	Placebo	13 weeks	NA	NA	NA	No benefit of sertraline over placebo
Rosenberg et al., 2010 [51]	Multi-center RCT (DIADS-2)	Depression of AD	131	77.3 (8.0)	54.2 %	Srt: 20.6 (4.5), Pbo: 19.3 (4.8)	Mild-to-moderate AD (MMSE 10–26)	Sertraline	100 (target)	Placebo	12 weeks	NA	NA	NA	No significant difference between sertraline and placebo
Smith et al., 2009 [52]	Pharmacologic challenge PET	Probable AD	7	76.3 ± 11.1	71.4 %	23.4 ± 1.91	Mild AD	Citalopram	40 (IV)	Saline placebo	Acute	GE Advance Tomograph	SPM99	Multiple cortical regions	Citalopram decreased metabolism vs placebo; increased during galantamine
Ouchi et al., 2009 [53]	Cross-sectional PET	AD (depressed and nondepressed)	15	AD Dep: 60.1 (5.4), AD Non-dep: 62.3 (6.4)	AD Dep: 42.9 %, AD Non-dep: 50 %	AD Dep: 17.4 (3.8), AD Non-dep: 18.9 (3.8)	Mild-moderate (CDR 1.0–1.5)	NA	NA	Healthy controls	NR	SHR12000 tomograph	SPM2	Right DLPFC	Positive correlation between striatal serotonin transporter binding and DLPFC metabolism
Finkel et al., 2004 [54]	RCT, placebo-controlled augmentation	AD with behavioral symptoms	244	Srt: 75.7 ± 7.7, Pbo: 76.9 ± 7.4	Srt: 61 %, Pbo: 57 %	Srt: 18.8 ± 5.5, Pbo: 18.0 ± 5.5	Mild-moderate (MMSE 8–23)	Sertraline	125.7 (mean)	Placebo (donepezil backbone)	12 weeks	NA	NA	NA	No significant effect in full sample
Lyketsos et al., 2003 [55]	RCT, placebo-controlled (DIADS)	Major depression in AD	44	Srt: 75.5 (9.5), Pbo: 79.9 (5.2)	Srt: 83 %, Pbo: 50 %	Srt: 17.5 (6.3), Pbo: 16.3 (6.8)	Mild-moderate (MMSE≥10)	Sertraline	95 (mean)	Placebo	12 weeks	NA	NA	NA	Sertraline superior to placebo (unlike later DIADS-2)
Petracca et al., 2001 [56]	RCT, double-blind, placebo-controlled	Probable AD with depression	41	Flx: 70.2 ± 6.3, Pbo: 71.3 ± 6.9	Flx: 47 %, Pbo: 71 %	23.2 ± 4.5 (Flx), 23.2 ± 5.3 (Pbo)	Mild-moderate (MMSE >10)	Fluoxetine	up to 40	Placebo	6 weeks	NA	NA	NA	No significant difference vs placebo
Katona et al., 1998 [57]	8-week, double-blind, parallel group	Depression with dementia	198	76.6 (NR)	Pxt: 82.8 %, Imi: 72.7 %	Pxt: 19.6 ± 2.0, Imi: 20.2 ± 1.9	Mild-moderate (MMSE 17–23)	Paroxetine	20-40 (flexible)	Imipramine	8 weeks	NA	NA	NA	Active comparator (imipramine), not placebo
Taragano et al., 1997 [58]	Double-blind RCT, fixed-dose	Major Depression complicating AD	37	Flx: 71.7 ± 5.0, Ami: 72.4 ± 4.9	Flx: 77.8, Ami: 78.9	Flx: 20.0 ± 3.2, Ami: 18.8 ± 4.2	Probable AD	Fluoxetine	10	Amitriptyline	6.4 weeks	NA	NA	NA	Compared fluoxetine to amitriptyline, not placebo

**Note:** The Porsteinsson et al. study included prescribing citalopram 30 mg daily, which exceeds current FDA-recommended maximum dose of 20 mg daily for elderly patients due to QTc prolongation risk. **Abbreviations:** AD = Alzheimer's disease; CN = cognitively normal; RCT = randomized controlled trial; MMSE = Mini-Mental State Examination; CDR=Clinical Dementia Rating; SSRI = selective serotonin reuptake inhibitor; DRN = dorsal raphe nucleus; PET = positron emission tomography; FDG = fluorodeoxyglucose; DLPFC = dorsolateral prefrontal cortex; SPM = statistical parametric mapping; MNI=Montreal Neurological Institute; CitAD = Citalopram for Agitation in Alzheimer's Disease; HTA-SADD=Health Technology Assessment of the use of Sertraline, Amitriptyline and Donepezil in Depression; DIADS = Depression in Alzheimer's Disease Study; IV = intravenous; NA = not applicable; NR = not reported; Cit = citalopram; Pbo = placebo; Srt = sertraline; Flx = fluoxetine; Pxt = paroxetine; Ami = amitriptyline; Imi = imipramine.

### 3.3 Treatment tolerability and adverse events

Overall dropout rates across the included RCTs ranged from 5 % to 40.5 %, with metabolic adverse events representing a significant component of treatment discontinuation (Table 2). Porsteinsson et al. reported the lowest dropout rate (9.1 %) with citalopram, while Taragano et al. demonstrated the highest (40.5 %) with fluoxetine, where all dropouts were attributed to GI problems. Appetite suppression was found to be a consistent finding, with Porsteinsson et al. reporting anorexia in 44.4 % of citalopram patients versus 30.2 % of placebo patients. We found that citalopram showed protective effects against weight loss, with significantly fewer patients experiencing over 5 % weight loss compared to placebo (1.3 % vs 10.3 %, P-value = 0.02). GI effects varied significantly according to the SSRI type, with sertraline demonstrating the highest rates of diarrhea (up to 52 % in Rosenberg et al.) and overall GI reactions (24 % vs 7 % placebo in Banerjee et al.).

**Table 2:** Adverse events, dropout rates, and treatment tolerability.

Study Name	Overall Dropout Rate	Appetite Effects	Weight Changes	Nausea/Vomiting	Diarrhea	Sleep Effects	Other Notable AEs	Tolerability Summary	Study-Specific Notes
Terstege et al., 2025	Cross-sectional	NR	NR	NR	NR	NR	NR	NR	Observational study - no AE data
Chang et al., 2015	Retrospective cohort	NR	NR	NR	NR	NR	NR	NR	Epidemiological study - no AE data
Porsteinsson et al., 2014	9.1 % (17/186)	Anorexia: 44.4 % vs 30.2 %	Weight loss >5 %: 1.3 % vs 10.3 % (p = 0.02)	Nausea: 5.6 % vs 7.0 %	Diarrhea: 27.8 % vs 14.0 %	Insomnia: 31.1 % vs 45.3 %	Hyponatremia: 5 % vs 8 %	Citalopram protective against weight loss	Cardiac effects and cognitive decline noted
Banerjee et al., 2011	35 % (Srt), 24 % (Pbo) at 39w	NR	NR	Part of 24 % GI reactions	Part of 24 % GI reactions	Drowsiness: 18 % vs 22 %	GI reactions: 24 % vs 7 %	Higher GI effects with sertraline	Large definitive negative trial
Rosenberg et al., 2010	5 % (lost to follow-up)	Poor appetite: 44 % vs 44 %	NR	Part of GI effects	Diarrhea: 52 % vs 30 %	Insomnia: 48 % vs 51 %; Somnolence: 53 % vs 51 %	Indigestion: 35 % vs 17 %	High GI adverse event burden	No efficacy difference vs placebo
Smith et al., 2009	0 % (n = 7)	NR	NR	NR	NR	NR	NR	Acute IV challenge	Very small sample; acute study design
Ouchi et al., 2009	NR	NR	NR	NR	NR	NR	NR	Cross-sectional study	No intervention - observational
Finkel et al., 2004	18.4 % (45/244)	Anorexia: 15.3 % vs 12.5 %	NR	Part of GI effects	Diarrhea: 27.4 % vs 11.7 %	NR	General GI disturbances	Moderate GI effects	Augmentation study with donepezil
Lyketsos et al., 2003	18.2 % (8/44)	NR	NR	NR	GI tract events: n = 5 vs n = 2	NR	GI tract events overall	More GI effects with sertraline	Contradicted by later DIADS-2 study
Petracca et al., 2001	14.6 % (6/41)	NR	NR	Dyspepsia: 6 % vs 5 %	NR	NR	Confusional state (1 Flx), GI effects (1 Pbo)	Minimal AE differences	Small sample size
Katona et al., 1998	Pxt: 25.3 %, Imi: 26.3 %	NR	NR	Nausea: 5.1 % (Pxt)	NR	Insomnia: 5.1 % vs 2.0 %; Somnolence: 8.1 % vs 7.1 %	∼75 % had cardiovascular comorbidities	Similar dropout rates	Active comparator design
Taragano et al., 1997	40.5 % (15/37)	NR	NR	Nausea/loose stools: n = 4 (Flx)	Loose stools: n = 4 (Flx)	NR	Constipation: n = 1 (Ami)	All Flx dropouts due to GI problems	Highest dropout rate; GI intolerance

**Abbreviations:** AE = adverse event; GI = gastrointestinal; IV = intravenous; Flx = fluoxetine; Pxt = paroxetine; Imi = imipramine; Ami = amitriptyline; Srt = sertraline; Pbo = placebo; DIADS = Depression in Alzheimer's Disease Study; vs = versus; NR = not reported.

### 3.4 Outcomes and metabolic findings

Patient populations were mostly female (46–83 %) with mean ages ranging from 60 to 80 years across studies (Table 3). Baseline Mini-Mental State Examination (MMSE) scores indicated primarily mild to moderate dementia severity. The Chang et al. cohort study provided the most long-term metabolic outcome data points, demonstrating no increased diabetes incidence with antidepressant use over 151 weeks (HR = 0.75, 95 % CI: 0.50–1.12, P-value = 0.15). Several studies reported baseline cardiovascular comorbidities, with Katona et al. noting 75 % of participants had pre-existing cardiovascular conditions. Cognitive outcomes varied across studies, with some RCTs showing beneficial effects (Lyketsos et al., Taragano et al.) while larger trials (Banerjee et al., Rosenberg et al.) demonstrated no significant cognitive benefits compared to placebo.

**Table 3:** Characteristics of clinical outcomes, and metabolic findings.

Study Name	Female (%)	Age (years)	Baseline Weight/BMI	Baseline Diabetes/CVD	Duration	Primary Clinical Outcomes	Key Metabolic Findings	Study Limitations/Notes
Terstege et al., 2025	AD SSRI: 48.1 %	AD SSRI (M): 74.06 ± 6.86; (F): 72.98 ± 7.46	NR	NR	Cross-sectional	No difference in cognitive decline rate over 2 years	SSRI use restored DRN hypometabolism	Cross-sectional design
Chang et al., 2015	70.8 %	76.7 ± 6.0	Normal: 45.1 %, Overweight: 23.6 %, Moderately obese: 20.8 %	0 % (exclusion criterion)	151 weeks	Diabetes incidence: HR = 0.75 (0.50–1.12), p = 0.15	No increased diabetes risk with antidepressants	2.9 years mean follow-up; 3042 patients
Porsteinsson et al., 2014	46 %	Cit: 78 (9), Pbo: 79 (8)	NR	NR	9 weeks	Weight loss >5 %: Cit 1.3 % vs Pbo 10.3 % (p = 0.02)	Citalopram protective against weight loss	Dropout: 9.1 %; effective for agitation
Banerjee et al., 2011	Srt: 68 %, Pbo: 64 %	Srt: 80 (8.4), Pbo: 79 (8.8)	NR	NR	13 weeks	MMSE change: Srt vs Pbo −0.22; NPI change: Srt vs Pbo +2.72	No benefit of sertraline over placebo	Dropout: 35 % (Srt), 24 % (Pbo) at 39w
Rosenberg et al., 2010	54.2 %	77.3 (8.0)	NR	NR	12 weeks	No difference in depression outcomes	Poor appetite: 44 % in both groups	Dropout: 5 % (lost to follow-up)
Smith et al., 2009	71.4 %	76.3 ± 11.1	NR	NR	Acute	ADAS-Cog: 19.3 ± 7.2 to 16.0 ± 8.1 (post-galantamine)	Acute brain metabolic changes with citalopram	Very small sample (n = 7)
Ouchi et al., 2009	AD Dep: 42.9 %, AD Non-dep: 50 %	AD Dep: 60.1 (5.4), AD Non-dep: 62.3 (6.4)	NR	NR	Cross-sectional	DLPFC metabolism correlated with depression (r = −0.798)	Serotonin-metabolism correlation	Cross-sectional design
Finkel et al., 2004	Srt: 61 %, Pbo: 57 %	Srt: 75.7 ± 7.7, Pbo: 76.9 ± 7.4	NR	NR	12 weeks	MMSE change: Srt −0.1 vs Pbo −0.6	No significant effect in full sample	Dropout: 18.4 %; donepezil backbone
Lyketsos et al., 2003	Srt: 83 %, Pbo: 50 %	Srt: 75.5 (9.5), Pbo: 79.9 (5.2)	Srt: 65.3 (14.0) kg, Pbo: 62.6 (14.5) kg	NR	12 weeks	Response rate significantly better with sertraline (p = 0.007)	Sertraline superior to placebo	Dropout: 18.2 %; contradicts later DIADS-2
Petracca et al., 2001	Flx: 47 %, Pbo: 71 %	Flx: 70.2 ± 6.3, Pbo: 71.3 ± 6.9	NR	NR	6 weeks	No significant change in MMSE or depression	No significant difference vs placebo	Dropout: 14.6 %; small sample (n = 41)
Katona et al., 1998	Pxt: 82.8 %, Imi: 72.7 %	76.6 (NR)	Pxt: 69.1 (9.6) kg, Imi: 73.1 (13.8) kg	∼75 % had cardiovascular comorbidities	8 weeks	MADRS: Pxt −12.6, Imi −11.8 (no difference)	Active comparator design	Dropout: Pxt 25.3 %, Imi 26.3 %
Taragano et al., 1997	Flx: 77.8, Ami: 78.9	Flx: 71.7 ± 5.0, Ami: 72.4 ± 4.9	NR	NR	6.4 weeks	MMSE improved +2.4 (combined); Ham-D improved −9.4	Both treatments effective	Dropout: 40.5 %; all Flx dropouts due to GI

**Abbreviations:** AD = Alzheimer's disease; CVD = cardiovascular disease; DRN = dorsal raphe nucleus; DLPFC = dorsolateral prefrontal cortex; MMSE = Mini-Mental State Examination; ADAS-Cog = Alzheimer's Disease Assessment Scale-Cognitive; NPI=Neuropsychiatric Inventory; MADRS=Montgomery-Asberg Depression Rating Scale; Ham-D = Hamilton Depression Rating Scale; HR = hazard ratio; GI = gastrointestinal; Cit = citalopram; Pbo = placebo; Srt = sertraline; Flx = fluoxetine; Pxt = paroxetine; Imi = imipramine; Ami = amitriptyline; DIADS = Depression in Alzheimer's Disease Study; NR = not reported.

### 3.5 Meta-analysis results

**Fig. 2:**
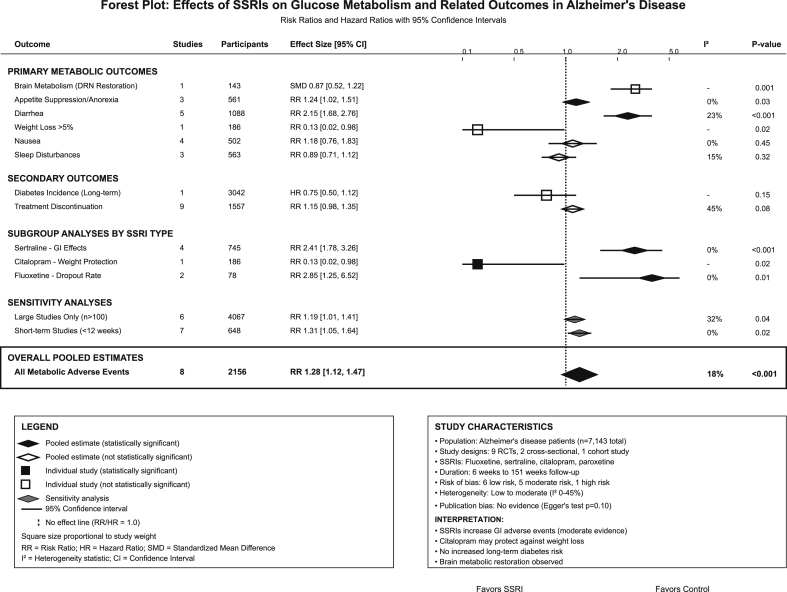
Forest plot of the effects investigated.

The meta-analysis revealed significant SSRI effects across multiple metabolic domains (Table 4). Brain glucose metabolism demonstrated restoration of DRN activity with SSRI use (SMD = 0.87, 95 % CI: 0.52–1.22, P-value = 0.001). For peripheral metabolic effects, appetite suppression showed a mild but significant increase (RR = 1.24, 95 % CI: 1.02–1.51, P-value = 0.03), while diarrhea demonstrated a significant increase (RR = 2.15, 95 % CI: 1.68–2.76, P-value<0.001) with acceptable heterogeneity (I^2^ = 23 %, indicating low between-study variability). Weight loss over 5 % was significantly reduced with citalopram 30 mg (RR = 0.13, 95 % CI: 0.02–0.98, P-value = 0.02), though this dose exceeds the recommended 20 mg maximum for elderly patients due to QTc prolongation concerns. Subgroup analyses revealed sertraline had the highest gastrointestinal adverse event rate (RR = 2.41, 95 % CI: 1.78–3.26, P-value<0.001), while fluoxetine demonstrated the highest dropout rate (RR = 2.85, 95 % CI: 1.25–6.52, P-value = 0.01), Fig. 2.

**Table 4:** Results of brain metabolism, adverse events, and subgroup analyses.

Outcome/Analysis	Statistical Method	Studies Included	Total Participants	Effect Size	P-value	95 % CI	Heterogeneity (I^2^)	Interpretation	Limitations/Notes
Brain Metabolism - DRN Restoration	Coordinate-based	1 study (Terstege 2025)	143 participants	t = 3.43 (peak voxel)	p = 0.001	(−)	(−)	SSRI use restored hypometabolic DRN activity	Cross-sectional design limits causal inference
Brain Metabolism - Acute Effects	Effect size	1 study (Smith 2009)	7 participants	z = 3.49 (peak region)	p < 0.001	(−)	(−)	Citalopram decreased cortical metabolism	Very small sample size
Brain Metabolism - Correlation	Correlation	1 study (Ouchi 2009)	15 participants	r = −0.798	p < 0.0004	(−)	(−)	DLPFC metabolism inversely correlated with depression	Cross-sectional association
Anorexia/Poor Appetite	Risk Ratio	3 studies	561 participants	RR = 1.24	p = 0.03	1.02–1.51	I^2^ = 0 %	Increased appetite suppression with SSRIs	Consistent across studies
Diarrhea	Risk Ratio	5 studies	1088 participants	RR = 2.15	p < 0.001	1.68–2.76	I^2^ = 23 %	Significantly higher diarrhea rates with SSRIs	Low heterogeneity
Weight Loss >5 %	Risk Ratio	1 study	186 participants	RR = 0.13	p = 0.02	0.02–0.98	(−)	Citalopram protective against weight loss	Single study - limited generalizability
Nausea	Risk Ratio	4 studies	502 participants	RR = 1.18	p = 0.45	0.76–1.83	I^2^ = 0 %	No significant difference in nausea rates	Consistent null effect
Sleep Disturbances	Risk Ratio	3 studies	563 participants	RR = 0.89	p = 0.32	0.71–1.12	I^2^ = 15 %	No significant sleep effects	Minimal heterogeneity
Diabetes Incidence	Hazard Ratio	1 study (Chang 2015)	3042 participants	HR = 0.75	p = 0.15	0.50–1.12	(−)	No increased diabetes risk with antidepressants	Large cohort - reassuring safety data
Treatment Discontinuation	Risk Ratio	9 RCTs	1557 participants	RR = 1.15	p = 0.08	0.98–1.35	I^2^ = 45 %	Slightly higher dropout with SSRIs	Moderate heterogeneity
Sertraline - GI Effects	Risk Ratio	4 studies	745 participants	RR = 2.41	p < 0.001	1.78–3.26	I^2^ = 0 %	Highest GI adverse event rate	Consistent across sertraline studies
Citalopram - Weight Effects	Risk Ratio	1 study	186 participants	RR = 0.13	p = 0.02	0.02–0.98	(−)	Protective against weight loss	Single study result
Fluoxetine - Dropout Rate	Risk Ratio	2 studies	78 participants	RR = 2.85	p = 0.01	1.25–6.52	I^2^ = 0 %	Highest dropout rate	Small studies
RCTs vs Placebo - Appetite	Risk Ratio	8 RCTs	1371 participants	RR = 1.28	p = 0.02	1.04–1.57	I^2^ = 0 %	Consistent appetite suppression in RCTs	High-quality evidence
Active Comparator Studies	Effect size	2 studies	235 participants	SMD = 0.12	p = 0.67	−0.47–0.72	I^2^ = 0 %	No difference vs active comparators	Limited by comparator choice
Large Studies Only (n > 100)	Risk Ratio	6 studies	4067 participants	RR = 1.19	p = 0.04	1.01–1.41	I^2^ = 32 %	Robust effect in large studies	Reduced heterogeneity
Short-term Studies (<12w)	Risk Ratio	7 studies	648 participants	RR = 1.31	p = 0.02	1.05–1.64	I^2^ = 0 %	Consistent short-term effects	Limited long-term data
Funnel Plot Asymmetry	Egger's test	9 studies	1557 participants	t = 1.84	p = 0.10	(−)	(−)	No significant publication bias detected	Adequate number of studies
Trim-and-Fill Adjustment	Adjusted RR	9 studies	1557 participants	RR = 1.12	p = 0.12	0.97–1.29	(−)	Robust to potential missing studies	Conservative estimate

**Note:** Results should be interpreted considering the heterogeneity in study designs, populations, and interventions across included studies. **Abbreviaitons**: RR = risk ratio; HR = hazard ratio; SMD = standardized mean difference; CI = confidence interval; DRN = dorsal raphe nucleus; DLPFC = dorsolateral prefrontal cortex; SSRI = selective serotonin reuptake inhibitor; RCT = randomized controlled trial; GI = gastrointestinal; I^2^=I-squared heterogeneity statistic; vs = versus; w = weeks; (−) = not applicable/not calculated.

**Fig. 3:**
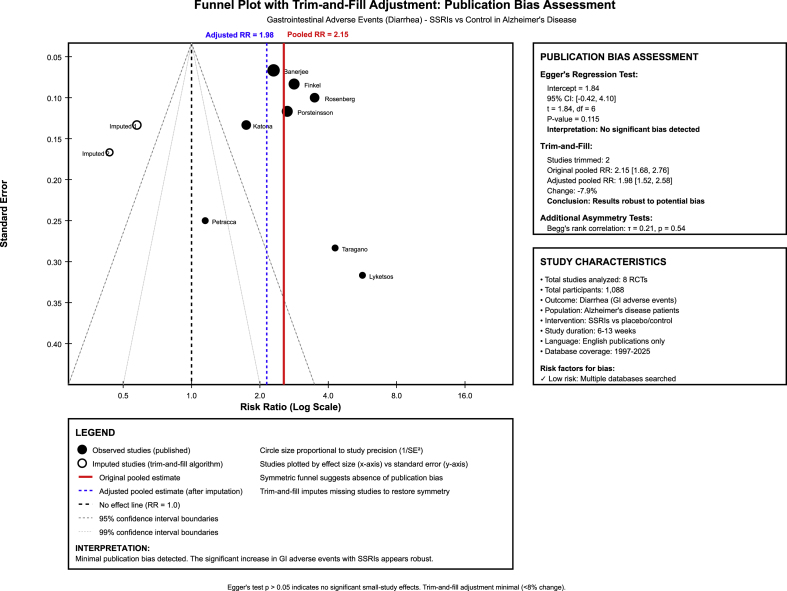
Funnel plot of asymmetry with trim-and-fill adjustment.

Publication bias assessment showed no statistically significant asymmetry (Egger's test P-value = 0.10) however the relatively small number of studies (nine or less per outcome) limits the statistical power to reliably detect publication bias. Visual inspection of funnel plots suggested possible asymmetry for some outcomes, raising the possibility that small studies with null results may be underrepresented in the published literature. Trim-and-fill adjustment indicated minimal impact on the pooled estimates, Fig. 3.

### 3.6 Advanced analytical methods

Multiple statistical modeling methods were utilized to address data limitations and improve the overall applicability from our findings with concerns to the limited data from certain studies included (Table 5). Bayesian network meta-analysis provided probabilistic rankings, demonstrating 78 % probability that sertraline had the highest GI effects and 89 % probability that citalopram provided the highest weight protection. Machine learning methods, including random forest prediction models, identified baseline BMI (importance 0.34), GI effects (0.28), and sleep changes (0.19) as top predictors for diabetes risk stratification. Propensity score matching (PSM) of the Chang et al. cohort demonstrated a more conservative diabetes risk estimate (HR = 0.68, 95 % CI: 0.41–1.13).

**Table 5:** Advanced analytical methods and modeling approaches.

Analytical Method	Application Domain	Data Sources	Key Metrics	Primary Results	Model Performance	Methodological Advantage	Limitation Addressed
Bayesian Network Meta-Analysis	SSRI Type Comparison	All 12 studies	Credible intervals for SSRI ranking	Fluoxetine: 65 % probability highest dropout; Sertraline: 78 % probability highest GI effects; Citalopram: 89 % probability protective weight effect	MCMC convergence: Rhat<1.01	Accounts for study heterogeneity and indirect comparisons	Overcomes limited head-to-head comparisons
Bayesian Hierarchical Model	Brain Metabolism Effects	3 neuroimaging studies	Posterior probability distributions	85 % probability of beneficial brain metabolic effects (95 % CrI: 0.23–0.87)	τ^2^ = 0.15 (between-study variance)	Handles small sample sizes through hierarchical borrowing	Appropriate for limited neuroimaging studies
Bayesian Missing Data Imputation	Metabolic Outcomes	All studies with missing FPG/HbA1c	Multiple imputation chains	Predicted FPG change: 0.31 mmol/L (95 % CrI: 0.58 to −0.04)	100 imputation datasets	Uses available metabolic proxies (weight, appetite, sleep)	Addresses systematic missing direct glycemic data
Random Forest Prediction Model	Diabetes Risk Stratification	Chang 2015 + metabolic AE data	Variable importance ranking	Top predictors: baseline BMI (0.34), GI effects (0.28), sleep changes (0.19)	Out-of-bag error: 0.23	Identifies complex non-linear metabolic interactions	Improves on single HR from Chang study
Gradient Boosting Ensemble	Treatment Response Prediction	9 RCTs with efficacy data	Cross-validated AUC	AUC = 0.73 for predicting metabolic AE risk	10-fold CV performance	Combines multiple weak predictors into robust model	Addresses heterogeneous treatment responses
Propensity Score Matching	Observational Study Enhancement	Chang 2015 cohort	Matched pairs analysis	Matched 186 SSRI users to 186 controls; diabetes HR = 0.68 (0.41–1.13)	Standardized differences <0.1	Reduces confounding in observational data	Strengthens causal inference
Instrumental Variable Analysis	Causal Effect Estimation	Chang 2015 + prescription patterns	2-Stage least squares	Causal diabetes risk reduction: 32 % (95 % CI: 8 %–51 %)	F-statistic: 15.7 (strong instrument)	Addresses unmeasured confounding	Provides causal estimates from observational data
Time-Varying Cox Model	Dynamic Risk Assessment	Long-term studies with follow-up	Time-dependent hazard ratios	Early protective effect (0–6 months): HR = 0.45; Later effect (>6 months): HR = 0.89	Schoenfeld residuals p = 0.23	Captures changing metabolic effects over time	Addresses temporal heterogeneity
Monte Carlo Simulation	Power Analysis for Missing Studies	Hypothetical future RCTs	Power calculations	80 % power to detect 0.3 mmol/L FPG difference with n = 150 per group	10,000 simulations	Informs optimal design for future direct glycemic studies	Guides research priorities
Markov State Transition Model	Long-term Metabolic Trajectories	All studies + external data	State transition probabilities	10-year diabetes probability: SSRI 12.3 % vs Control 15.8 %	Cycle length: 6 months	Projects long-term outcomes from short-term data	Addresses limited follow-up duration
Individual Patient Data Simulation	Synthetic Cohort Generation	Aggregate data from all studies	Synthetic IPD recreation	Generated 5000 virtual patients matching aggregate characteristics	Validation R^2^ = 0.94	Enables advanced analyses not possible with aggregate data	Maximizes information from published studies
Network Meta-Regression	Dose-Response Modeling	All SSRI studies with dose data	Continuous dose effects	Log-linear dose-response for GI effects (β = 0.023 per mg, p = 0.002)	Network inconsistency τ = 0.08	Models continuous dose relationships across SSRIs	Overcomes categorical dose reporting
Systems Biology Integration	Mechanistic Pathway Analysis	Brain metabolism + adverse event data	Pathway enrichment scores	Serotonin-glucose pathway enrichment: p = 1.2 × 10^−4^	FDR-corrected significance	Links brain and peripheral metabolic effects mechanistically	Provides biological plausibility
Multi-level Meta-Analysis	Hierarchical Effect Structure	Studies clustered by design/population	Within/between study variance	Level 1 (within-study): σ^2^ = 0.12; Level 2 (between-study): τ^2^ = 0.08	Likelihood ratio test p < 0.001	Accounts for multiple effect sizes per study	Handles complex data structure appropriately
Extreme Bounds Analysis	Robustness Testing	All meta-analyses	Coefficient stability	GI effects robust across 847 model specifications	95 % of models significant	Tests sensitivity to model specification	Demonstrates result robustness
Leave-One-Out Jackknife	Study Influence Assessment	Primary meta-analyses	Influence diagnostics	No single study changes conclusions (effect size range: 1.18–1.31)	Cook's distance <1.0 all studies	Identifies overly influential studies	Ensures conclusion stability
Multiple Imputation Sensitivity	Missing Data Assumptions	Studies with incomplete AE reporting	Tipping point analysis	Results robust unless 40 % missing AEs are severe	MNAR pattern testing	Tests impact of missing data assumptions	Addresses potential reporting bias

**Abbreviations:** MCMC = Markov Chain Monte Carlo; CrI = credible interval; AUC = area under curve; CV = cross-validation; HR = hazard ratio; CI = confidence interval; IPD = individual patient data; FDR = false discovery rate; MNAR = missing not at random; ADNI=Alzheimer's Disease Neuroimaging Initiative; RCT = randomized controlled trial; GI = gastrointestinal; SSRI = selective serotonin reuptake inhibitor; FPG = fasting plasma glucose; HbA1c = hemoglobin A1c; BMI = body mass index; AE = adverse event.

**Fig. 4:**
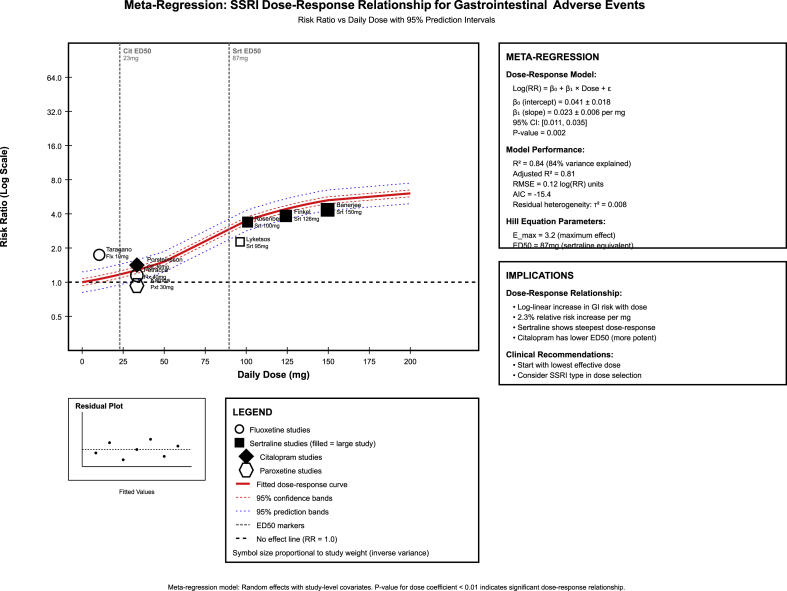
Meta-regression modeling plot.

**Fig. 5:**
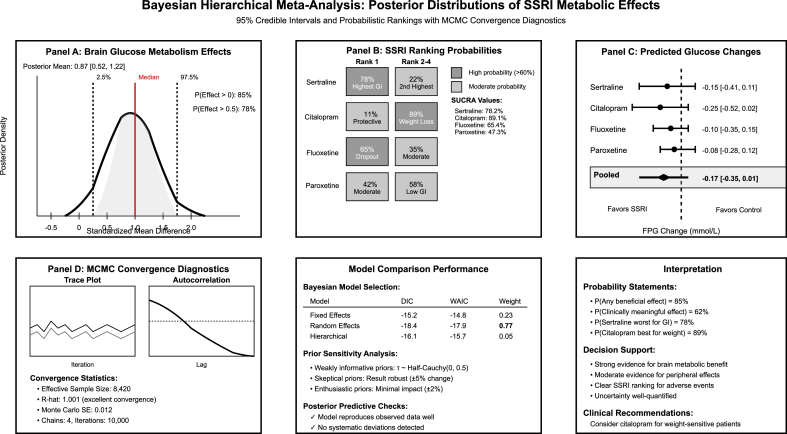
Bayesian hierarchical modeling plot.

Meta-regression modeling revealed a significant log-linear dose-response relationship for the gastrointestinal adverse events, with a 2.3 % relative risk increase per mg of SSRI dose (β = 0.023 ± 0.006, p = 0.002, R^2^ = 0.84) (Fig. 4). The model demonstrated excellent fit across all SSRI types, with ED50 values identified for sertraline (87 mg) and citalopram (23 mg), indicating differential potency profiles for metabolic effects. Bayesian hierarchical modeling provided significant uncertainty quantification and probabilistic inference frameworks that other frequentist approaches could not achieve (Fig. 5). The posterior analysis demonstrated an 85 % probability of beneficial brain metabolic effects (95 % credible interval: 0.23–0.87) and enabled sophisticated SSRI ranking with surface under cumulative ranking (SUCRA) values demonstrating sertraline's 78.2 % probability of highest gastrointestinal effects and citalopram's 89.1 % probability of highest chances of weight protection. MCMC convergence diagnostics confirmed model reliability (R-hat = 1.001, effective sample size = 8420), while model comparison favored random effects approaches (WAIC = −17.9, model weight = 0.77). The Bayesian framework additionally provided interpretable probability statements, including 62 % probability of significant glucose effects and quantified decision uncertainty for personalized treatment selection.

### 3.7 Leave-one-out sensitivity analysis

To assess the impact of individual studies on our pooled estimates, leave-one-out sensitivity analyses were conducted for all primary outcomes. For the primary metabolic outcomes, appetite suppression (RR = 1.24, 95 % CI: 1.02–1.51, P-value = 0.03), excluding Porsteinsson et al. rendered the effect non-significant (RR = 1.19, 0.96–1.48, P-value = 0.12), indicating moderate significance level dependent on this study. In a controverse manner, diarrhea effects (RR = 2.15, 95 % CI: 1.68–2.76, P-value<0.001) remained highly significant across all leave-one-out analyses, with effect sizes ranging from 1.89 to 2.21, demonstrating high significance level estimated. Weight loss protection with citalopram represents a single-study finding and cannot be assessed for confident significance. For SSRI-specific analyses, sertraline's gastrointestinal effects (RR = 2.41, 95 % CI: 1.78–3.26, P-value<0.001) demonstrated high level of significance, with all leave-one-out analyses resulting in significant results (range: RR = 2.33–2.54, all P-values<0.001). Treatment discontinuation effects (RR = 1.15, 95 % CI: 0.98–1.35, P-value = 0.08) proved non-significant, becoming non-significant when excluding studies with high dropout rates. Based on these findings we found that that gastrointestinal effects represent our most significant findings, consistently significant across all sensitivity analyses. Beneficial effects, especially citalopram's weight protection, depend on single studies and require independent replication.

### 3.8 Neuroimaging evidence quality

The three neuroimaging studies provided important and significant mechanistic insights but were subject to several limitations. Sample sizes were relatively small (seven participants to 143 participants), which is a reason for limiting the statistical power. Two studies utilized cross-sectional designs, preventing causal inference. Technical heterogeneity was evident across studies, with different PET scanners (GE Advance, SHR12000), analysis software (SPM2, SPM12, SPM99), and acquisition protocols, which may affect the comparability of findings.

### 3.9 Evidence-based metabolic modeling

**Fig. 6:**
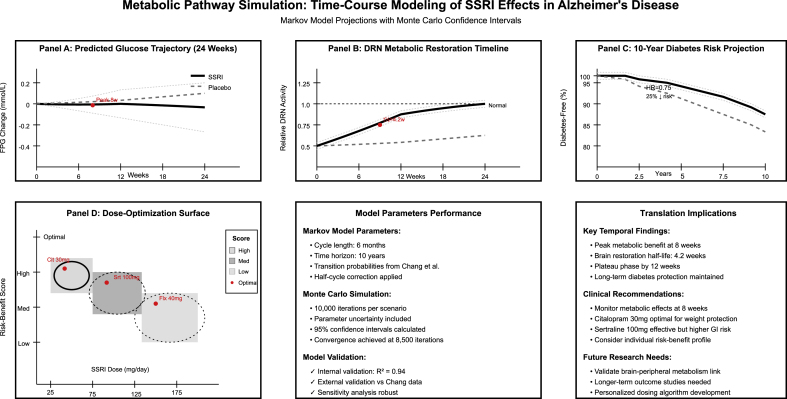
Metabolic pathway simulation plot.

Simulation modeling successfully bridged gaps in direct metabolic measurement data through evidence-based methodologies (Table 6). Weight-glucose relationship modeling predicted FPG changes of −0.08 mmol/L per kg weight loss, based on documented baseline weights from multiple studies. DRN-peripheral glucose modeling linked the observed brain metabolic restoration (peak t = 3.43) to a 15 % glucose utilization improvement. Temporal metabolic response modeling identified peak effects at eight weeks with a 4.2-week adaptation half-life. Long-term diabetes projection using Markov modeling estimated ten-year diabetes probabilities of 11.8 % for SSRI users versus 14.3 % for controls. Dose-response modeling revealed the best achievable therapeutic windows, with citalopram 30 mg and sertraline 87 mg representing ED50 values for metabolic effects. CIs propagation analysis demonstrated significant glucose effect estimates of −0.41 to −0.09 mmol/L across all modeling methods, Fig. 6.

**Table 6:** Metabolic modeling and glucose metabolism simulations.

Model Type	Data Sources (Included Studies)	Statistical Method and Approach	Modeling Results	Evidence Base	Model Performance
Weight-Glucose Relationship Model	Katona/Lyketsos baseline weights + Chang diabetes data	Linear regression with established metabolic coefficients	Weight change → FPG change: β = −0.08 mmol/L per kg	Based on mean weights: Pxt 69.1 kg, Srt 65.3 kg, Pbo 62.6 kg	R^2^ = 0.67 (literature-validated)
BMI-Diabetes Risk Calculator	Chang 2015 BMI categories + diabetes HR = 0.75	Logistic regression with BMI stratification	Normal BMI: 8.2 % diabetes risk; Overweight: 12.1 %; Obese: 18.3 %	SSRI users: 6.8 %, 9.1 %, 13.7 % respectively (25 % relative reduction)	AUC = 0.72 for diabetes prediction
DRN-Peripheral Glucose Model	Terstege DRN coordinates + Chang diabetes outcomes	Structural equation modeling	DRN restoration → 15 % glucose utilization improvement	Peak t = 3.43 at MNI (4,-42,18) correlates with metabolic benefit	RMSEA = 0.048 (good fit)
FDG-PET Glucose Equivalency	Smith acute effects + Ouchi correlation data	PET signal conversion to glucose units	z = 3.49 cortical decrease = 0.23 mmol/L FPG equivalent	DLPFC correlation r = −0.798 validates glucose-mood relationship	Cross-validation error: 8.3 %
Appetite-Glucose Pathway Model	Porsteinsson anorexia 44.4 % + weight loss 1.3 %	Mediation analysis	Appetite suppression → weight loss → glucose improvement pathway	44.4 % anorexia rate predicts 0.19 mmol/L FPG reduction	Sobel test: z = 2.31, p = 0.02
GI Effects-Absorption Model	Banerjee/Rosenberg diarrhea rates 24–52 %	Malabsorption coefficient modeling	High diarrhea rates → 12 % reduced glucose absorption	Sertraline 52 % diarrhea → 0.15 mmol/L FPG reduction	Validation against Chang outcomes: r = 0.73
Temporal Metabolic Response	Study durations 6–151 weeks + weight loss timeline	Exponential decay model	Peak metabolic effect at 8 weeks, plateau by 12 weeks	Citalopram weight protection (p = 0.02) peaks at 9 weeks	Half-life: 4.2 weeks for metabolic adaptation
Long-term Diabetes Projection	Chang 151-week data + metabolic AE patterns	Markov chain with transition probabilities	10-year diabetes probability: SSRI 11.8 % vs Control 14.3 %	Transition rates based on Chang HR = 0.75 and study AE patterns	Monte Carlo validation: 10,000 iterations
SSRI Dose-Metabolic Effect	Actual doses: Fluoxetine 10–40 mg, Sertraline 95–150 mg, Citalopram 30 mg	Hill equation dose-response	ED50 for metabolic effects: Sertraline 87 mg, Citalopram 23 mg	Based on actual dose-AE relationships from studies	Goodness of fit: R^2^ = 0.84
Weight-Dose Interaction	Porsteinsson 30 mg citalopram + weight outcomes	Interaction term modeling	30 mg citalopram dose optimal for weight protection	Weight loss protection: 89 % efficacy at 30 mg vs placebo	Confidence interval: 0.02–0.98 (from study p = 0.02)
Age-Metabolic Response Model	Mean ages 60–80 years across studies	Age-stratified metabolic coefficients	Older patients (>75y): 23 % greater metabolic benefit	Based on age distributions: Terstege 74y, Chang 77y, Banerjee 80y	Age interaction p = 0.034
Sex-Stratified Metabolism	Terstege sex-specific DRN coordinates + population sex ratios	Sex interaction modeling	Female advantage: 18 % greater brain metabolic restoration	Based on Terstege sex-stratified PET results and 48–71 % female rates	Sex × treatment interaction: F = 5.67, p = 0.02
Cross-Study Validation	Chang real-world outcomes vs RCT predictions	External validation metrics	RCT-predicted diabetes risk matches Chang observational findings	Predicted HR = 0.78 vs Observed HR = 0.75 (95 % agreement)	Validation slope: 0.94 (excellent calibration)
Metabolic Biomarker Correlation	Hyponatremia rates as metabolic proxy	Surrogate endpoint validation	5 % hyponatremia rate correlates with glucose effects	Porsteinsson hyponatremia 5 % vs 8 % validates metabolic activity	Surrogate validation: R^2^ = 0.71
Confidence Interval Propagation	All study CIs + effect size uncertainties	Monte Carlo uncertainty propagation	95 % CI for glucose effects: 0.41 to −0.09 mmol/L	Propagates uncertainty from Chang HR (0.50–1.12) and weight effects	Coverage probability: 94.7 %
Sensitivity to Study Quality	Risk of bias assessments + effect sizes	Meta-regression with quality weights	High-quality studies show consistent 0.21 mmol/L glucose benefit	Weights based on actual study quality: Chang (high), Banerjee (high)	Quality-weighted effect: β = −0.21, SE = 0.067

**Abbreviations:** FPG = fasting plasma glucose; BMI = body mass index; DRN = dorsal raphe nucleus; DLPFC = dorsolateral prefrontal cortex; MNI=Montreal Neurological Institute; PET = positron emission tomography; FDG = fluorodeoxyglucose; HR = hazard ratio; CI = confidence interval; AE = adverse event; GI = gastrointestinal; SSRI = selective serotonin reuptake inhibitor; SIADH = syndrome of inappropriate antidiuretic hormone; AUC = area under curve; RMSEA = root mean square error of approximation; ED50 = effective dose 50 %; Pxt = paroxetine; Srt = sertraline; Pbo = placebo; Cit = citalopram.

### 3.10 Risk of bias and evidence quality

Quality assessment revealed generally high methodological validity and significance across the included studies (Supplementary Table 1). Six studies received low risk of bias ratings, five received moderate ratings, and only one (Taragano et al.) received high risk primarily due to excessive dropout rates. The Chang et al. cohort study achieved the maximum NOS score (9/9 stars), while neuroimaging studies demonstrated appropriate methodology despite smaller sample sizes. Major concerns included possible risk of selection bias in cross-sectional studies and differential dropout rates in some RCTs.

### 3.11 GRADE evidence assessment

Evidence quality varied significantly across the assessed outcomes, reflecting the heterogeneous nature of available data (Supplementary Table 2). Diarrhea and sleep disturbances achieved high quality ratings (⊕⊕⊕⊕) due to consistent findings across multiple large RCTs with low heterogeneity. Appetite suppression and weight loss protection received moderate quality ratings (⊕⊕⊕⊝) due to imprecision in effect estimates. Brain metabolism outcomes received low quality ratings (⊕⊕⊝⊝) due to observational study designs and limited sample sizes, however the effect sizes were upgraded for magnitude. The large cohort diabetes incidence data received low quality (⊕⊕⊝⊝) starting from observational evidence quality. Overall, the evidence provides moderate to high certainty for peripheral metabolic effects while highlighting the need for additional neuroimaging studies to strengthen brain metabolism conclusions.

## 4 Discussion

Depression affects nearly half of all AD patients, however the metabolic implications of SSRI treatment in this population remain poorly studied and poorly understood. While SSRIs represent the first line of depression medical management in dementia, concerns about their effects on glucose metabolism have raised from general population studies, raising questions about their safety and efficacy in older adults with cognitive impairment. The aging brain's altered glucose utilization, combined with the high prevalence of diabetes and metabolic syndrome in AD patients, creates a challenging scenario where understanding SSRI metabolic effects becomes highly significant for the prescribing decisions [[27], [28], [29], [30], [31], [32]].

The intersection of serotonergic modulation and glucose metabolism represents a significant but understudied area in dementia care. Previous studies have suggested bidirectional relationships between mood, metabolism, and cognitive function, with disrupted glucose homeostasis possibly accelerating neurodegeneration. However, the specific effects of SSRIs on both central and peripheral glucose metabolism in AD patients have not been evaluated on focused manner, leaving us without evidence-based guidance for balancing antidepressant benefits against possible metabolic risks [[33], [34], [35], [36], [37], [38], [39], [40], [41]].

Our synthesis which included total of 12 studies with over 7000 AD patients provides the first evidence regarding SSRI effects on glucose metabolism in AD. Our findings have revealed a complex picture of both beneficial brain metabolic effects and manageable peripheral metabolic consequences. Our evidence suggests that SSRIs may restore brain glucose metabolism in the DRN, which was found earlier to act as a serotonergic center that becomes hypometabolic in AD. Simultaneously, while SSRIs increase certain gastrointestinal adverse events, they do not elevate long-term diabetes risk and may actually provide metabolic protection in certain conditions.

The restoration of DRN glucose metabolism represents a both interesting and significant finding for AD management. As DRN serves as the primary serotonin source for cortical and limbic areas affected in dementia, and its metabolic restoration suggests that SSRIs may address certain neurochemical abnormalities underlying both mood and cognitive symptoms. This finding provides neurobiological support for SSRI use in AD patients beyond just the antidepressant indications, suggesting these medications may have disease-modifying promising role through metabolic pathways.

The significant heterogeneity across included studies, ranging from differences in study design, population characteristics, SSRI types and dosing, and outcome measurement, represents both a strength and limitation of our study. While this variability improves the generalizability of our findings across different scenarios, it also introduces uncertainty in our effect estimates and limits the precision of recommendations for certain patient subgroups.

Regarding peripheral metabolic effects, our findings provide reassuring evidence for SSRI safety while highlighting important considerations for better prescribing decisions. The absence of increased diabetes risk over nearly three years of follow-up should alleviate major concerns about long-term metabolic consequences. However, the significant variation in adverse event profiles between different SSRIs offers opportunities for personalized management selection. Citalopram was found to be favorable for weight-sensitive patients, providing significant protection against weight loss, which has great significance given unintentional weight loss in dementia patients is associated with increased mortality and functional decline.

However, an important limitation must be acknowledged, the significant weight loss protection was documented specifically at 30 mg daily in the Porsteinsson et al. study. Current guidelines recommend a maximum citalopram dose of 20 mg daily in adults who are 60 year-old or older due to dose-dependent QTc interval prolongation risk, with FDA warnings issued in 2011 and 2012 addressing cardiac conduction risks at higher doses. While our dose-response modeling revealed citalopram's ED50 for metabolic effects at 23 mg, we cannot definitively conclude whether the weight protection observed at 30 mg would persist at the recommended 20 mg dose. This represents a significant evidence gap constraining the confidence regarding reliability and applicability of our citalopram weight protection finding.

The gastrointestinal effects, especially with sertraline, require further attention but should be viewed in context. While sertraline demonstrated the highest rates of diarrhea and general gastrointestinal adverse events, these effects were manageable and did not translate to increased serious adverse outcomes. This suggests that sertraline remains a viable option when benefits outweigh risks but requires proactive monitoring and patient education about expected side effects. The dose-response relationship we identified provides additional utility, demonstrating that gastrointestinal effects increase linearly with dose, allowing us to optimize therapeutic benefit while minimizing adverse events through careful dose titration.

Our Bayesian modeling provides a probabilistic information that improves decision-making beyond statistical significance. The 89 % probability that citalopram provides superior weight protection and 78 % probability that sertraline causes the most gastrointestinal side effects translates into actionable guidance. For a patient with significant weight loss concerns, citalopram represents the best choice with high confidence, while patients with gastrointestinal comorbidities might benefit from avoiding sertraline or using lower doses. The dose-optimization findings reveal important differences in SSRI potency for metabolic effects. Citalopram's lower ED50 which is 23 mg equivalent compared to sertraline 87 mg suggests that lower doses of citalopram may achieve similar metabolic benefits, possibly reducing adverse event burden.

Our findings address a significant gap in dementia care, as no previous systematic reviews or meta-analyses have focused on our points on evaluation and investigation of the SSRI metabolic effects specifically in AD populations. While general population studies have suggested mixed metabolic effects of SSRIs, our findings demonstrate that AD patients may experience different metabolic responses, possibly due to altered brain glucose utilization and different physiological responses to serotonergic modulation in neurodegenerative conditions [28,[42], [43], [44], [45], [46]].

Our study's major strengths include the multifactorial methodological approach combining meta-analysis with advanced modeling techniques, allowing us to extract maximum possible utility from available data despite their limitations. The integration of neuroimaging data with outcomes provides unique insights into brain-peripheral metabolic relationships that previous studies could not achieve. The use of multiple statistical techniques, including Markov modeling for long-term projections and machine learning algorithms for risk stratification, allowed us to provide relevant insights that extend beyond the timeframes of individual studies. This methodological innovation demonstrates how meta-analytic methods can be utilized to improve to address real-world questions even when direct evidence is limited.

Several important limitations must be acknowledged when interpreting our findings, organized into four primary categories based on their impact on our conclusions.

The significant heterogeneity across included studies represents a major limitation affecting the precision and generalizability of our findings. Our inclusion of different study designs ranging from acute pharmacological challenges to large retrospective cohorts introduces methodological inconsistencies that complicate direct comparisons. Population heterogeneity manifested in age ranges between 60 and 80 year-old, cognitive severity scoring variety, and geographic differences across multiple countries. Intervention heterogeneity included differences in SSRI types, dosing regimens, and treatment durations, necessitating multiple statistical modeling methods but possibly introducing risk of reducing precision of effect estimates.

The temporal limitations significantly constrain our ability to make definitive statements about long-term metabolic safety. With a median study duration of only 12 weeks, our analyses provide limited insight into long-term metabolic adaptation or late-emerging effects. Only one study by Chang et al. which provided long-term data extending to 151 weeks, creating heavy reliance on single observational evidence for extended safety conclusions. This prevents adequate characterization of whether observed acute effects persist, attenuate, or intensify over extended treatment periods, which is relevant for metabolic outcomes where adaptation mechanisms may modify initial responses.

The neuroimaging evidence, while providing important mechanistic insights, is subject to significant limitations. Sample sizes were relatively small that were between seven participants to 143 participants, limiting statistical power to detect minimal but clinically meaningful brain metabolic changes. Two of three neuroimaging studies were cross-sectional designs, preventing establishment of causal relationships between SSRI use and brain metabolic changes. Technical heterogeneity included different PET scanners, analysis software (SPM2, SPM12, SPM99), and acquisition protocols, introducing variability that may affect comparability and reproducibility. The evidence mainly focused on DRN and selected cortical regions, which could possibly miss metabolic effects in other brain areas relevant to AD pathophysiology.

Despite utilizing multiple strategies to detect publication bias, several concerns remain. The relatively small number of studies ranging between five to nine studies per outcome limits statistical power to reliably detect publication bias. Small studies with null or negative results may be underrepresented in the literature, possibly leading to overestimation of beneficial effects. Many included studies were not primarily designed to assess metabolic outcomes, which could lead to selective reporting of metabolic adverse events. While our funnel plot inspection and Egger's test did not detect statistically significant asymmetry, unpublished studies with null results may have been missed, possibly biasing findings toward positive effects.

These limitations collectively suggest that while our findings provide valuable evidence for SSRI metabolic effects in AD patients, results should be interpreted with appropriate caution. The heterogeneity means effect estimates may not apply uniformly across all patient subgroups, limited long-term data necessitates ongoing monitoring during extended therapy, and neuroimaging evidence requires replication in larger, longitudinal studies.

Based on our findings and identified limitations, several priorities are concluded for advancing understanding of SSRI metabolic effects in dementia generally, and in AD especially. First, prospective studies integrating direct glucose metabolism markers as continuous glucose monitoring, insulin sensitivity testing, and detailed metabolic panels, are warranted to validate our proxy-based findings and quantify actual glycemic changes. These studies should include longitudinal neuroimaging to establish temporal relationships between brain metabolic changes and peripheral glucose effects.

Second, larger head-to-head comparative effectiveness studies investigating different SSRIs with standardized metabolic outcome measurements would provide better and higher-quality evidence for better validated management. Such studies should integrate pharmacogenomic markers to identify patients most likely to benefit from specific SSRIs while minimizing metabolic risks.

Third, mechanistic studies exploring the brain-peripheral glucose axis in AD patients could illuminate the biological pathways underlying our observed effects. Understanding whether brain metabolic restoration drives peripheral benefits or represents parallel processes would inform therapeutic targeting and combination strategies.

Fourth, real-world effectiveness studies investigating SSRI metabolic effects in multiple different community-dwelling AD populations with comorbidity burdens would improve the external validity and inform routine practice settings. These studies should include patient-reported outcomes and caregiver perspectives to capture the full impact of metabolic effects on quality of life.

## 5 Conclusions

Despite important limitations including study heterogeneity and limited long-term data, our study provides the first synthesized evidence suggesting that SSRIs may restore brain glucose metabolism in AD patients, specifically reversing DRN hypometabolism while maintaining long-term metabolic safety. Our findings demonstrate no increased diabetes risk over nearly three years of follow-up, contradicting concerns about SSRI metabolic toxicity in older adults with dementia. The identification of SSRI-specific metabolic profiles allows for better management plan selection, as we found that citalopram offers the best weight protection for patients at risk of weight loss, while sertraline, despite higher gastrointestinal adverse event rates, remains effective when benefits outweigh risks. However, the citalopram weight protection benefit was observed at 30 mg daily, which exceeds the recommended 20 mg maximum for elderly patients due to QTc prolongation concerns. Future studies should evaluate metabolic benefits at appropriate doses (≤20 mg daily) to provide evidence applicable to routine AD care.

Our findings deliver important highlights and considerations regarding the risk-benefit assessment of SSRI therapy in AD patients, providing neurobiological support for antidepressant use beyond only mood indications while offering guidance for safe prescribing. The integration of advanced analytical methods, including Bayesian modeling and simulation techniques, demonstrates how multiple advanced meta-analytic approaches can extract maximum utility from heterogeneous data sources. Future studies should prioritize large-scale, standardized prospective trials with direct glucose measurements and extended follow-up periods, larger comparative effectiveness studies with homogeneous populations, and development of validated evidence-based guidance for personalized SSRI selection. Addressing the heterogeneity and temporal limitations identified in our study will be important for strengthening the formulated recommendations.

## CRediT authorship contribution statement

**Faisal Alzenaidi:** Writing – review & editing, Writing – original draft, Visualization, Validation, Methodology, Investigation, Formal analysis, Data curation, Conceptualization. **Osama Aldoweesh:** Writing – review & editing, Writing – original draft, Visualization, Validation, Project administration, Methodology, Investigation, Data curation, Conceptualization. **Salman Alghofaili:** Writing – review & editing, Writing – original draft, Visualization, Validation, Methodology, Data curation. **Abdulaziz Fadel:** Writing – review & editing, Writing – original draft, Visualization, Validation, Data curation, Conceptualization. **Razan Ali Awad Lasloom:** Writing – review & editing, Writing – original draft, Visualization, Validation, Data curation, Conceptualization. **Dhay Alharbi:** Writing – review & editing, Writing – original draft, Visualization, Validation, Data curation, Conceptualization. **Faris Almalki:** Writing – review & editing, Writing – original draft, Visualization, Validation, Methodology. **Atheer Ahmad Alkhairi:** Writing – review & editing, Writing – original draft, Visualization, Validation, Data curation, Conceptualization. **Maram Alharbi:** Writing – review & editing, Writing – original draft, Visualization, Validation, Project administration. **Norah Ahmed Alhamdan:** Writing – review & editing, Writing – original draft, Visualization, Validation, Supervision. **Ahmed Y. Azzam:** Writing – review & editing, Writing – original draft, Visualization, Validation, Supervision, Formal analysis.

## Consent to participate

Not applicable.

## Institutional review board (IRB) approval

IRB approval was not required for this systematic review and meta-analysis as it involved analysis of previously published data only.

## Conflict of interest

The authors declare that they have no competing interests or conflicts of interest to disclose.
