# Supplementary material for: Selective serotonin reuptake inhibitors and glucose metabolism in Alzheimer's disease and related dementias: A systematic review and meta-analysis of brain metabolic and adverse event data
Source: Metabol Open. 2025 Aug 28;27:100389. doi: 10.1016/j.metop.2025.100389 (PMC12423674; doi:10.1016/j.metop.2025.100389)
Supplement: Multimedia component 1 [file mmc1.docx]

**Supplementary Table 1:** Risk of Bias Assessment.

| **Study** | **Design** | **Assessment Tool** | **Domain 1** | **Domain 2** | **Domain 3** | **Overall Score** | **Risk of Bias** | **Rationale and Comments** |
| --- | --- | --- | --- | --- | --- | --- | --- | --- |
| Terstege et al. 2025 | Cross-sectional | Newcastle-Ottawa Scale | Selection: ★★★☆ (3/4) | Comparability: ★★ (2/2) | Outcome: ★★☆ (2/3) | 7/9 stars | Moderate | ADNI data source reliable; limited by cross-sectional design and potential selection bias for SSRI users |
| Chang et al. 2015 | Retrospective cohort | Newcastle-Ottawa Scale | Selection: ★★★★ (4/4) | Comparability: ★★ (2/2) | Outcome: ★★★ (3/3) | 9/9 stars | Low | Large sample, well-defined cohort, appropriate follow-up; excellent methodological quality |
| Porsteinsson et al. 2014 | RCT | Cochrane RoB 2.0 | Randomization: Low risk | Deviations: Low risk | Missing data: Low risk (9.1% dropout) | Outcome measurement: Low risk | Low | Well-conducted RCT with appropriate randomization, blinding, and outcome assessment |
| Banerjee et al. 2011 | RCT | Cochrane RoB 2.0 | Randomization: Low risk | Deviations: Low risk | Missing data: Some concerns (35% dropout) | Outcome measurement: Low risk | Low | Large, well-designed RCT; high dropout rate at extended follow-up but ITT analysis performed |
| Rosenberg et al. 2010 | RCT | Cochrane RoB 2.0 | Randomization: Low risk | Deviations: Low risk | Missing data: Low risk (5% dropout) | Outcome measurement: Low risk | Low | Multi-center RCT with excellent retention and outcome assessment |
| Smith et al. 2009 | Experimental PET | Modified NOS + Neuroimaging | Selection: ★★☆☆ (2/4) | Comparability: ★☆ (1/2) | Outcome: ★★★ (3/3) | 6/9 stars | Moderate | Very small sample (n=7); rigorous PET methodology but limited generalizability |
| Ouchi et al. 2009 | Cross-sectional | Newcastle-Ottawa Scale | Selection: ★★★☆ (3/4) | Comparability: ★☆ (1/2) | Outcome: ★★★ (3/3) | 7/9 stars | Moderate | Good PET methodology; limited by small sample and cross-sectional design |
| Finkel et al. 2004 | RCT | Cochrane RoB 2.0 | Randomization: Low risk | Deviations: Low risk | Missing data: Low risk (18.4% dropout) | Outcome measurement: Low risk | Low | Augmentation study with donepezil backbone; appropriate methodology and analysis |
| Lyketsos et al. 2003 | RCT | Cochrane RoB 2.0 | Randomization: Low risk | Deviations: Low risk | Missing data: Low risk (18.2% dropout) | Outcome measurement: Low risk | Low | Well-conducted pilot RCT; appropriate design and execution |
| Petracca et al. 2001 | RCT | Cochrane RoB 2.0 | Randomization: Low risk | Deviations: Low risk | Missing data: Low risk (14.6% dropout) | Outcome measurement: Low risk | Moderate | Small sample size (n=41) limits power; otherwise well-conducted |
| Katona et al. 1998 | RCT | Cochrane RoB 2.0 | Randomization: Low risk | Deviations: Low risk | Missing data: Some concerns (25% dropout) | Outcome measurement: Some concerns | Moderate | Active comparator design (vs imipramine); high dropout rate and potential detection bias |
| Taragano et al. 1997 | RCT | Cochrane RoB 2.0 | Randomization: Some concerns | Deviations: Some concerns | Missing data: High risk (40.5% dropout) | Outcome measurement: Some concerns | High | Very high dropout rate; all fluoxetine dropouts due to GI effects suggesting potential bias |

***Abbreviations:*** *RoB=Risk of Bias; NOS=Newcastle-Ottawa Scale; RCT=randomized controlled trial; PET=positron emission tomography; ITT=intention-to-treat; GI=gastrointestinal; ADNI=Alzheimer's Disease Neuroimaging Initiative; ★=star (quality indicator).*
