# Supplementary material for: Selective serotonin reuptake inhibitors and glucose metabolism in Alzheimer's disease and related dementias: A systematic review and meta-analysis of brain metabolic and adverse event data
Source: Metabol Open. 2025 Aug 28;27:100389. doi: 10.1016/j.metop.2025.100389 (PMC12423674; doi:10.1016/j.metop.2025.100389)
Supplement: Multimedia component 2 [file mmc2.docx]

**Supplementary Table 2:** GRADE Evidence Assessment.

| **Outcome** | **Studies (Participants)** | **Initial Quality** | **Risk of Bias** | **Inconsistency** | **Indirectness** | **Imprecision** | **Publication Bias** | **Large Effect** | **Dose-Response** | **Confounding** | **Final Quality** | **Summary** |
| --- | --- | --- | --- | --- | --- | --- | --- | --- | --- | --- | --- | --- |
| Brain glucose metabolism restoration (DRN) | 1 cross-sectional study (143 participants) | Low¹ | Not downgraded | Not downgraded | Downgraded (-1)⁴ | Not downgraded | Not assessed | Not upgraded | Not upgraded | Not upgraded | ⊕⊕⊝⊝ LOW | SSRI use may restore brain glucose metabolism in the dorsal raphe nucleus |
| Acute brain metabolism changes | 1 experimental study (7 participants) | Low¹ | Downgraded (-1)² | Not downgraded | Seriously downgraded (-2)⁵ | Not downgraded | Not assessed | Not upgraded | Not upgraded | Not upgraded | ⊕⊝⊝⊝ VERY LOW | Very uncertain about acute effects of SSRIs on brain glucose metabolism |
| Brain metabolism-depression correlation | 1 cross-sectional study (15 participants) | Low¹ | Not downgraded | Not downgraded | Seriously downgraded (-2)⁵ | Not downgraded | Not assessed | Upgraded (+1)⁶ | Not upgraded | Not upgraded | ⊕⊕⊝⊝ LOW | There may be a strong correlation between brain glucose metabolism and depression severity |
| Appetite suppression/anorexia | 3 RCTs (561 participants) | High | Not downgraded | Not downgraded | Not downgraded | Downgraded (-1)⁷ | Not assessed | Not upgraded | Not upgraded | Not upgraded | ⊕⊕⊕⊝ MODERATE | SSRIs probably increase appetite suppression compared to placebo |
| Diarrhea | 5 RCTs (1088 participants) | High | Not downgraded | Not downgraded | Not downgraded | Not downgraded | Not assessed | Not upgraded | Upgraded (+1)⁸ | Not upgraded | ⊕⊕⊕⊕ HIGH | SSRIs increase diarrhea rates compared to placebo |
| Weight loss >5% | 1 RCT (186 participants) | High | Not downgraded | Not downgraded | Not downgraded | Downgraded (-1)⁹ | Not assessed | Not upgraded | Not upgraded | Not upgraded | ⊕⊕⊕⊝ MODERATE | Citalopram probably reduces risk of significant weight loss |
| Nausea | 4 RCTs (502 participants) | High | Not downgraded | Not downgraded | Not downgraded | Not downgraded | Not assessed | Not upgraded | Not upgraded | Not upgraded | ⊕⊕⊕⊕ HIGH | SSRIs do not significantly increase nausea compared to placebo |
| Sleep disturbances | 3 RCTs (563 participants) | High | Not downgraded | Not downgraded | Not downgraded | Not downgraded | Not assessed | Not upgraded | Not upgraded | Not upgraded | ⊕⊕⊕⊕ HIGH | SSRIs do not significantly affect sleep compared to placebo |
| Diabetes incidence | 1 cohort study (3042 participants) | Low¹ | Not downgraded | Not downgraded | Not downgraded | Not downgraded | Not assessed | Not upgraded | Not upgraded | Not upgraded | ⊕⊕⊝⊝ LOW | SSRIs may not increase long-term diabetes risk |
| Treatment discontinuation | 9 RCTs (1557 participants) | High | Downgraded (-1)³ | Downgraded (-1)¹⁰ | Not downgraded | Not downgraded | Not assessed | Not upgraded | Not upgraded | Not upgraded | ⊕⊕⊝⊝ LOW | SSRIs may slightly increase treatment discontinuation |
| Sertraline GI effects | 4 RCTs (745 participants) | High | Not downgraded | Not downgraded | Not downgraded | Not downgraded | Not assessed | Not upgraded | Upgraded (+1)⁸ | Not upgraded | ⊕⊕⊕⊕ HIGH | Sertraline increases GI adverse events with large effect size |
| Age-related metabolic effects | Multiple studies (age 60-80) | High | Not downgraded | Not downgraded | Downgraded (-1)¹¹ | Downgraded (-1)¹² | Not assessed | Not upgraded | Not upgraded | Not upgraded | ⊕⊕⊝⊝ LOW | Age may modify metabolic effects of SSRIs |
| Sex-stratified brain metabolism | 1 study with sex analysis (143 participants) | Low¹ | Not downgraded | Not downgraded | Downgraded (-1)⁴ | Not downgraded | Not assessed | Not upgraded | Not upgraded | Not upgraded | ⊕⊕⊝⊝ LOW | Sex differences in brain metabolic response may exist |

***Reasons for Downgrading: ¹*** *Observational study design (starts at low quality)****; ²*** *Risk of bias: Very small sample size limits reliability****; ³*** *Risk of bias: High dropout rate in some studies (Taragano 40.5%)****; ⁴*** *Imprecision: Wide confidence intervals due to limited sample size****; ⁵*** *Imprecision: Very wide confidence intervals, very small sample size****; ⁷*** *Imprecision: Confidence interval includes null effect (RR 1.02-1.51)****; ⁹*** *Imprecision: Single study evidence, wide confidence interval****; ¹⁰*** *Inconsistency: Moderate heterogeneity (I²=45%) between studies****; ¹¹*** *Indirectness: Indirect evidence from subgroup analyses****; ¹²*** *Imprecision: Limited power for interaction testing* ***Abbreviaitons:*** *GRADE=Grading of Recommendations Assessment, Development and Evaluation; RCT=randomized controlled trial; DRN=dorsal raphe nucleus; SSRI=selective serotonin reuptake inhibitor; GI=gastrointestinal; RR=risk ratio; CI=confidence interval; I²=I-squared heterogeneity statistic.*
